# Short-term PM_2.5_ and cardiovascular admissions in NY State: assessing sensitivity to exposure model choice

**DOI:** 10.1186/s12940-021-00782-3

**Published:** 2021-08-23

**Authors:** Mike Z. He, Vivian Do, Siliang Liu, Patrick L. Kinney, Arlene M. Fiore, Xiaomeng Jin, Nicholas DeFelice, Jianzhao Bi, Yang Liu, Tabassum Z. Insaf, Marianthi-Anna Kioumourtzoglou

**Affiliations:** 1 Department of Environmental Health Sciences, Columbia University Mailman School of Public Health, New York, NY USA; 2 Department of Environmental Medicine and Public Health, Icahn School of Medicine At Mount Sinai, One Gustave L. Levy Place, Box 1057, New York, NY 10029 USA; 3 Department of Environmental Health, Boston University School of Public Health, Boston, MA USA; 4 Department of Earth and Environmental Sciences, Columbia University, New York, NY USA; 5 Lamont-Doherty Earth Observatory, Columbia University, Palisades, NY USA; 6 Department of Chemistry, University of California, Berkeley, Berkeley, CA USA; 7 Department of Environmental & Occupational Health Sciences, University of Washington School of Public Health, Seattle, WA USA; 8 Gangarosa Department of Environmental Health, Emory University, Rollins School of Public Health, Atlanta, GA USA; 9 New York State Department of Health, Albany, NY USA; 10 School of Public Health, University At Albany, Rensselaer, NY USA

**Keywords:** Particulate matter, Exposure assessment, Cardiovascular morbidity

## Abstract

**Background:**

Air pollution health studies have been increasingly using prediction models for exposure assessment even in areas without monitoring stations. To date, most studies have assumed that a single exposure model is correct, but estimated effects may be sensitive to the choice of exposure model.

**Methods:**

We obtained county-level daily cardiovascular (CVD) admissions from the New York (NY) Statewide Planning and Resources Cooperative System (SPARCS) and four sets of fine particulate matter (PM_2.5_) spatio-temporal predictions (2002–2012). We employed overdispersed Poisson models to investigate the relationship between daily PM_2.5_ and CVD, adjusting for potential confounders, separately for each state-wide PM_2.5_ dataset.

**Results:**

For all PM_2.5_ datasets, we observed positive associations between PM_2.5_ and CVD. Across the modeled exposure estimates, effect estimates ranged from 0.23% (95%CI: -0.06, 0.53%) to 0.88% (95%CI: 0.68, 1.08%) per 10 µg/m^3^ increase in daily PM_2.5_. We observed the highest estimates using monitored concentrations 0.96% (95%CI: 0.62, 1.30%) for the subset of counties where these data were available.

**Conclusions:**

Effect estimates varied by a factor of almost four across methods to model exposures, likely due to varying degrees of exposure measurement error. Nonetheless, we observed a consistently harmful association between PM_2.5_ and CVD admissions, regardless of model choice.

**Supplementary Information:**

The online version contains supplementary material available at 10.1186/s12940-021-00782-3.

## Introduction

The association between air pollution and adverse health is one of the most well-researched topics in epidemiology, with studies spanning different pollutants [1–3], timescale of exposure [4–6], and outcomes of interest [7–9]. Historically, time-series studies in air pollution epidemiology have primarily utilized data from monitoring stations for exposure assignment. In the United States, this is primarily accomplished using data from the Environmental Protection Agency’s (EPA) Air Quality System (AQS) database [10]. Although monitors provide information on pollutant concentrations, there are strong assumptions when working with such data for health studies. For example, Bell et al. [11] noted that because the location of monitor systems also depends on regulatory compliance and not solely on population density, depending on the pollutant, monitor data are not necessarily best suited for public health research. Furthermore, monitor locations are by definition points in space and, thus, may not adequately capture population exposures in a pre-specified area in the time series analysis (e.g., a city) [12, 13].

To reduce exposure measurement error and, further, include populations in areas without monitors, there has been an increasing use of prediction models in air pollution epidemiology for exposure assessment. These prediction models provide outputs with full coverage at a much finer spatial resolution than a spatial point, which monitors represent, and predict both spatial and temporal changes in air pollution. Initially, such models were simple, and early efforts used largely statistical approaches, such as land use regression models and generalized additive mixed models [14–16]. With increased computation capacity and demand for higher spatial and temporal resolution, the prediction models have grown increasingly sophisticated. Examples include the integration of remote sensing data, predictions from chemical transport models, and more robust methods for higher predictive accuracy (e.g., random forests, neural networks, and ensemble models) [17–20].

Many research groups are currently developing and improving prediction models for exposure assessment in epidemiologic studies. However, most epidemiologic studies to date use air pollution predictions from a single model to assign exposures, although in recent years there have been additional efforts to develop statistical and computational exposure models with exhaustive datasets [21, 22]. This is of critical importance because the results from these epidemiologic studies are often used to inform regulations, but the exposure–response functions that are generated from studies using different models for exposure assessment are not necessarily comparable, both spatially and temporally. Our study aims to address this critical knowledge gap by assessing the sensitivity of fine particle (PM_2.5_; particles with aerodynamic diameter ≤ 2.5 µm) health effect estimates to the choices of different models for exposure assessment in a time series setting. As a case study, we focus on the association between daily PM_2.5_ concentrations and cardiovascular disease (CVD)-related hospitalizations in New York State (NYS) using a daily time series design at the county level. The goal of this paper is not to identify the “best” PM_2.5_ product, which would depend on the specific goals of a particular research project. Rather, we aim to characterize the potential variability in the results of epidemiologic analyses by using different PM_2.5_ products, and whether these results will allow us to reach similar or different conclusions in this NYS case study.

## Methods

### Exposure assessment

We obtained five publicly available, daily PM_2.5_ exposure products over NYS. These include data from the United States EPA’s AQS database, which provides PM_2.5_ monitoring data in 18 of 62 counties in NYS [10]; daily output from the Community Multiscale Air Quality Modeling System (CMAQ), an atmospheric chemical transport model developed by the EPA to simulate regional air pollution [23]; the Fused Air Quality Surface Using Downscaling (FAQSD), which uses a Bayesian space–time downscaler model to fuse the AQS measurements with CMAQ estimates [23]; a model developed by the United States Centers for Disease Control and Prevention’s Wide-ranging Online Data for Epidemiologic Research (CDC WONDER), which links satellite-derived and spatially interpolated ground-based PM_2.5_ using linear regression [24]; and a product from Emory University, which integrates satellite aerosol optical depth (AOD), land use data, and meteorological variables in a random forest model [19]. In Table 1 we present all of the PM_2.5_ datasets used in this study, with details regarding its spatial and temporal coverage and resolution. All PM_2.5_ datasets used in this study provided daily PM_2.5_ concentrations. More details regarding the PM_2.5_ products, including validation and comparative statistics, can be found in an existing publication [25].

**Table 1:** Summary of PM_2.5_ datasets used in this study

**Dataset**	**Short Name**	**Spatial Coverage**	**Spatial Resolution**	**Temporal Coverage**	**Reference**
US EPA Air Quality System^a^	AQS	USA	Point observations	1999–present	[10]
Community Multiscale Air Quality Modeling System	CMAQ	USA	12 × 12 km^2^	2002–2012	[23]
Fused Air Quality Surface using Downscaling	Fused	USA	12 × 12 km^2^	2002–2012	[23]
AQS and Remote Sensing Merged PM_2.5_^b^	CDC	USA	10 × 10 km^2^	2003–2011	[24]
Statistical Satellite-Based PM_2.5_	Emory	NYS	1 × 1 km^2^	2002–2012	[19]

^a^Monitoring sites were only available in 18 of the 62 counties in NYS

^b^Data were only available for 2003 – 2011

Exposure data were available from 2002 – 2012, except from the CDC WONDER model, which was available from 2003 – 2011. Daily average temperature and relative humidity were obtained from the North American Land Data Assimilation Systems (NLDAS), which provides the meteorological data at 1/8^th^ degree grids over the study area [26]. We averaged all available grids within a county to obtain daily county-level averages for all PM_2.5_ products and the corresponding meteorological variables.

### Outcome assessment

Daily total cardiovascular hospital admission counts for each county were obtained from the New York Department of Health’s Statewide Planning and Research Cooperative System (SPARCS). SPARCS is a comprehensive data reporting system that collects information on hospital admissions and emergency department visits within NYS, and includes approximately 98% of all hospitalizations in non-federal acute care facilities, regardless of insurance status [27]. The International Classification of Diseases, 9^th^ revision (ICD-9) was used to classify cardiovascular hospitalizations (ICD-9 codes 390 – 459).

Columbia University Institutional Review Board approval was obtained to conduct the analysis. The same board waived the need for informed consent because of the public nature of the data.

### Statistical analysis

This study is a daily time series analysis conducted at the county level. We employed overdispersed Poisson regression models to investigate the relationship between total CVD-related hospitalizations and same day PM_2.5_ exposure, separately for each set of PM_2.5_ concentrations, using all available data. In the models, we included smooth functions of calendar time to adjust for seasonality and long-term trends (using natural cubic splines with 4 *df* per year), as well as indicator variables for day of the week. To control for potential confounding by factors varying across counties, we included indicator variables for all counties used in the analyses (fixed effects). We controlled for potential confounding by weather by including smooth functions for daily average temperature (natural spline, 3 *df*) and relative humidity (natural spline, 3 *df*) in all models.

We selected the best fitting model and appropriate *df*s for all non-linear terms included in the model based on the quasi-Akaike Information Criterion (qAIC). Specifically, we tested for calendar time *df* from 4 to 7 per year, and for temperature, and relative humidity, from 3 to 6 *df*. We also assessed for potential nonlinearity in all PM_2.5_ products using a natural spline with 3 *df* and selected the model with the best fit using qAIC. For all PM_2.5_ products, the linear model yielded a better fit, so we only present results from the linear PM_2.5_ models.

We first ran analyses with all available information for each exposure model. To ensure comparability across all PM_2.5_ products, we then restricted the analysis to only the 18 counties where AQS data were available (“AQS only”), and finally to a dataset only with overlapping observations across all exposure datasets (“complete-case analysis”). We recognize that the results from this last set of analyses may have limited generalizability; however, our aim with this last analysis was to facilitate direct comparison across models. To maximize spatial coverage when comparing products, we performed a sensitivity analysis of the last model on a subset excluding AQS. Additionally, we also performed sensitivity analyses using the average of the same day and the previous day’s PM_2.5_ (lag 0–1) as the exposure window of interest, as well as adding federal holidays as a potential confounder.

It is likely that different exposure models perform differently in space and time. To assess the impact of varying prediction model performance in space and time, thus, in a secondary analysis we evaluated potential spatio-temporal effect modification using all available data. To assess effect modification by urban density, we obtained data on the urban and rural populations by county in NYS from the 2010 United States Census, and included in each model an interaction term between PM_2.5_ and number of individuals living in rural areas within each county. For effect modification varying by season, we broke each year up into four 3-month increments to define seasons: spring (March – May), summer (June – August), autumn (September – November), and winter (December – February) and included interaction terms between PM_2.5_ and season. We assessed statistical significance of the continuous interaction term (rural population) directly in the model, and of the interaction with the categorical season variable using a likelihood ratio test by comparing it to a model without the interaction term with season.

We present all results in the main analysis as percentage change in CVD admission rates per 10 µg/m^3^ increase in PM_2.5_. To facilitate comparison across PM_2.5_ products, we also present the results of our primary analysis per interquartile range in the Supplement. All statistical analyses were performed using the R Statistical Software, version 3.6.1 (Foundation for Statistical Computing, Vienna, Austria).

## Results

**Fig. 1:**
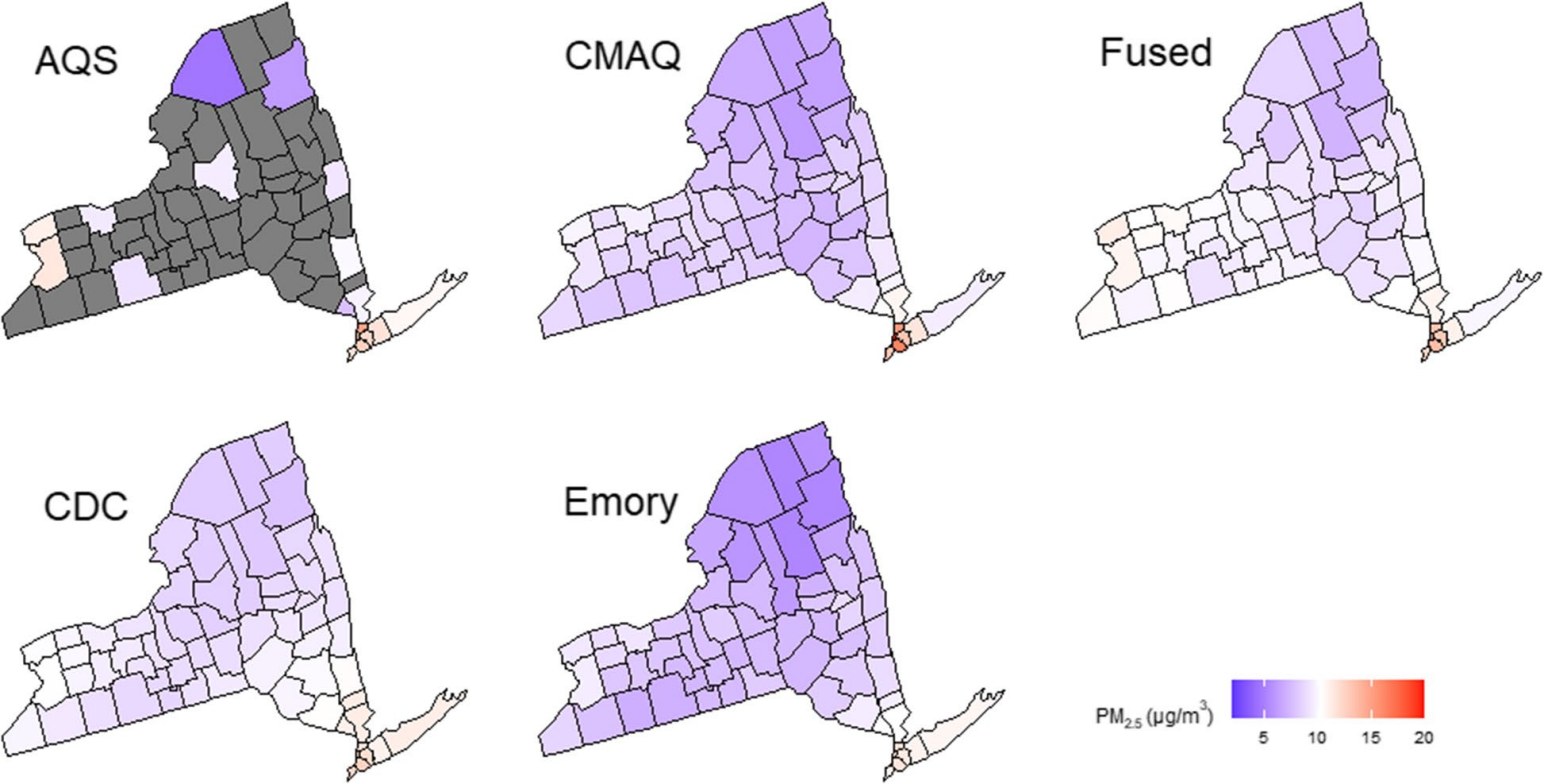
Spatial distributions of average PM_2.5_ exposure estimates by PM_2.5_ product

Figure 1 shows the PM_2.5_ county-wide exposure estimates by PM_2.5_ product averaged across the entire study period, and Table 2 shows the descriptive statistics of the variables used for the daily models for all available data. The average daily PM_2.5_ levels in the AQS, CMAQ, FAQSD, CDC WONDER, and Emory datasets were 10.7, 8.7, 9.8, 9.5, and 8.2 µg/m^3^, respectively. On average, 6.8 inpatient CVD admissions occurred per day and county. Descriptive statistics by season, quartiles of rural population, and those used for our complete-case analysis can be found in the Supplement (Tables S1–S3).

**Table 2:** Descriptive statistics for all 62 counties, unless otherwise noted (2002 – 2012)

**Variable**	**Mean**	**Min**	**25%**	**50%**	**75%**	**Max**	**% Missing**
Daily CVD admission counts per county	6.8	0.0	1.0	2.0	5.0	115.0	0.8
Daily PM_2.5_ (µg/m^3^)							
AQS^a^	10.7	0.0	6.0	9.1	13.6	97.1	75.7
CMAQ	8.7	0.0	4.2	6.9	11.4	98.2	0.0
Fused	9.8	0.2	5.6	8.2	12.3	99.7	0.3
CDC^b^	9.5	0.0	5.5	8.2	12.1	58.7	18.2
Emory	8.2	0.4	4.3	6.5	10.1	81.4	2.1
Mean temperature (°C)	9.0	-25.3	0.8	9.5	18.1	31.5	0.0
Relative humidity (%)	79.3	24.6	73.8	80.5	86.4	100.9	0.0

^a^Monitoring sites were only available in 18 of the 62 counties in NYS

^b^Data were only available for 2003–2011

Table 3 displays the correlation coefficients across the different PM_2.5_ products. In general, the AQS, Fused, CDC, and Emory products were all highly correlated with each other, with correlations ranging from 0.83 to 0.92. The CMAQ product, however, was moderately correlated with the other four products, with correlations only ranging from 0.49 to 0.61.

**Table 3:** Pairwise correlation coefficients across PM_2.5_ products for all available data

	AQS	CMAQ	Fused	CDC	Emory
AQS	1.00				
CMAQ	0.52	1.00			
Fused	0.89	0.61	1.00		
CDC	0.83	0.49	0.86	1.00	
Emory	0.90	0.52	0.92	0.85	1.00

**Fig. 2:**
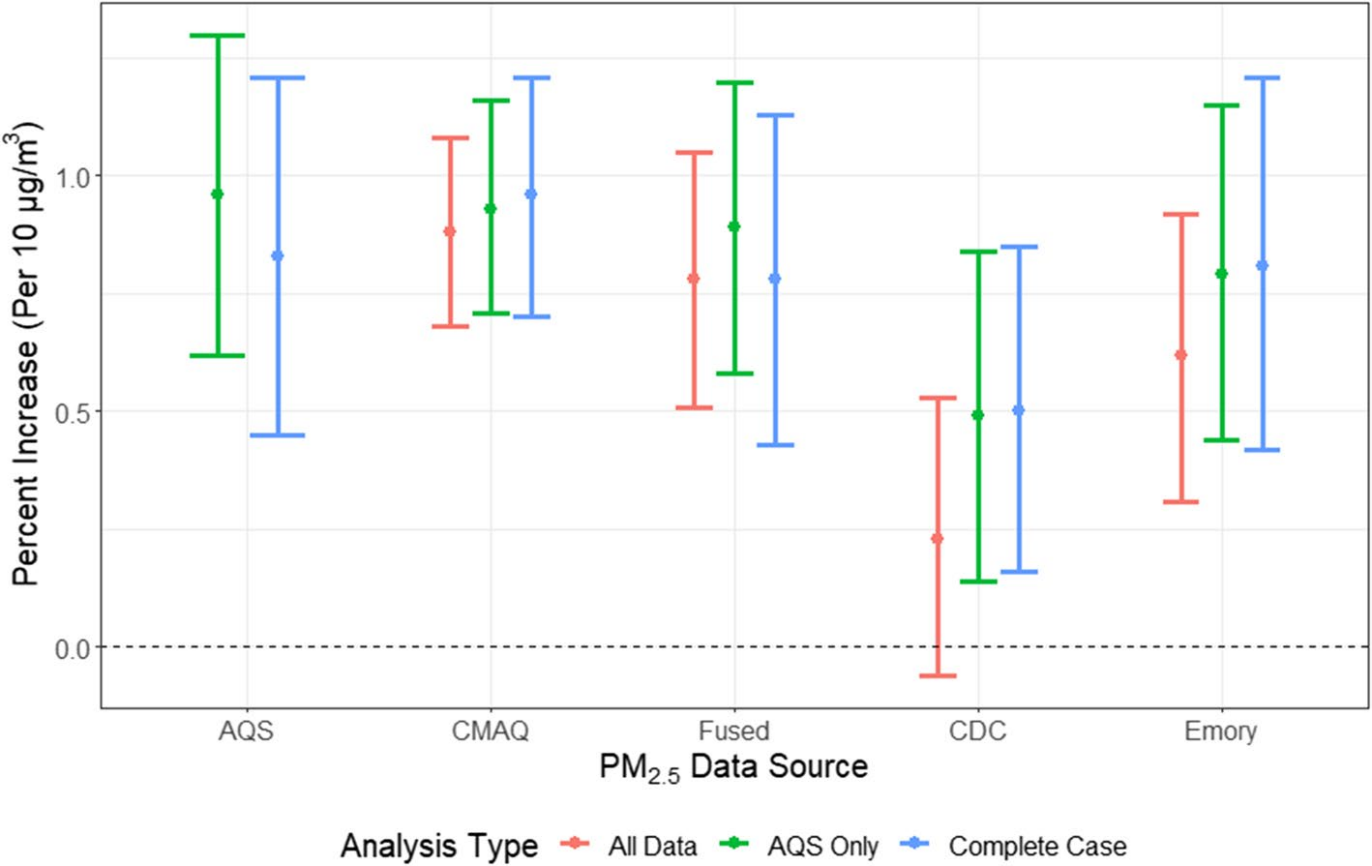
Percent increase in daily CVD admissions rates per 10 µg/m^3^ for all PM_2.5_ products. “All Data” refers to analyses using all available data for each exposure model from all 62 counties; “AQS Only” refers to analyses using data only in counties where AQS monitors were available (18 of 62 counties); “Complete Case” refers to analyses using data without any missingness across all five PM_2.5_ products

Figure 2 shows the percent change in CVD rate per 10 µg/m^3^ increase in PM_2.5_ across the different PM_2.5_ products and types of analyses. Effect estimates ranged from 0.23% (95%CI: -0.06, 0.53%) to 0.96% (95%CI: 0.70, 1.21%). Corresponding numeric estimates are presented in Table S4, and results showing the percent change in CVD rate per interquartile range increase in PM_2.5_ are shown in Figure S2. In general, we obtained the highest effect estimates with the tightest confidence intervals when we used CMAQ, while the CDC WONDER data yielded the lowest effect estimates. Our sensitivity analysis comparing effect estimates on overlapping observations across PM_2.5_ products excluding AQS and using average lag 0–1 PM_2.5_ exposure yielded very similar results to those obtained in the main analysis (Figures S2 and S3). Similarly, adding holidays as a potential confounder did not change our results (Figure S4).

**Fig. 3:**
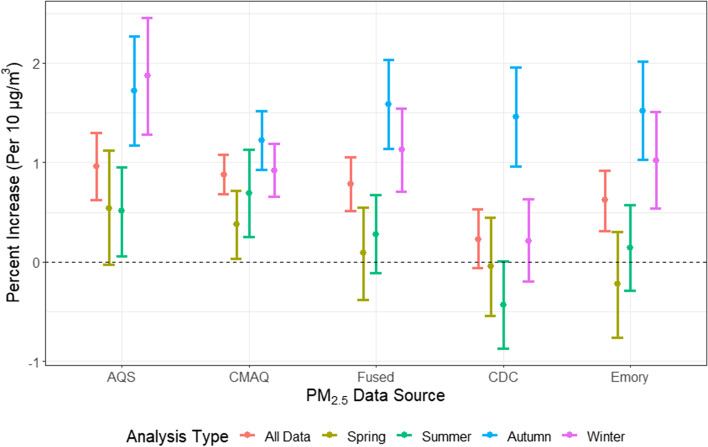
Effect modification by season using all available data

We detected evidence of effect modification by season for all PM_2.5_ products. Figure 3 shows the results assessing effect modification by season using all available data for each exposure model. For most products, we generally observed higher effect estimates in the autumn and winter seasons, reaching as high as a 1.87% increase in CVD admissions per 10 µg/m^3^ increase in PM_2.5_ in the autumn (for AQS). In comparison, the lowest effect estimates were observed in the spring, some of which were even negative.

**Fig. 4:**
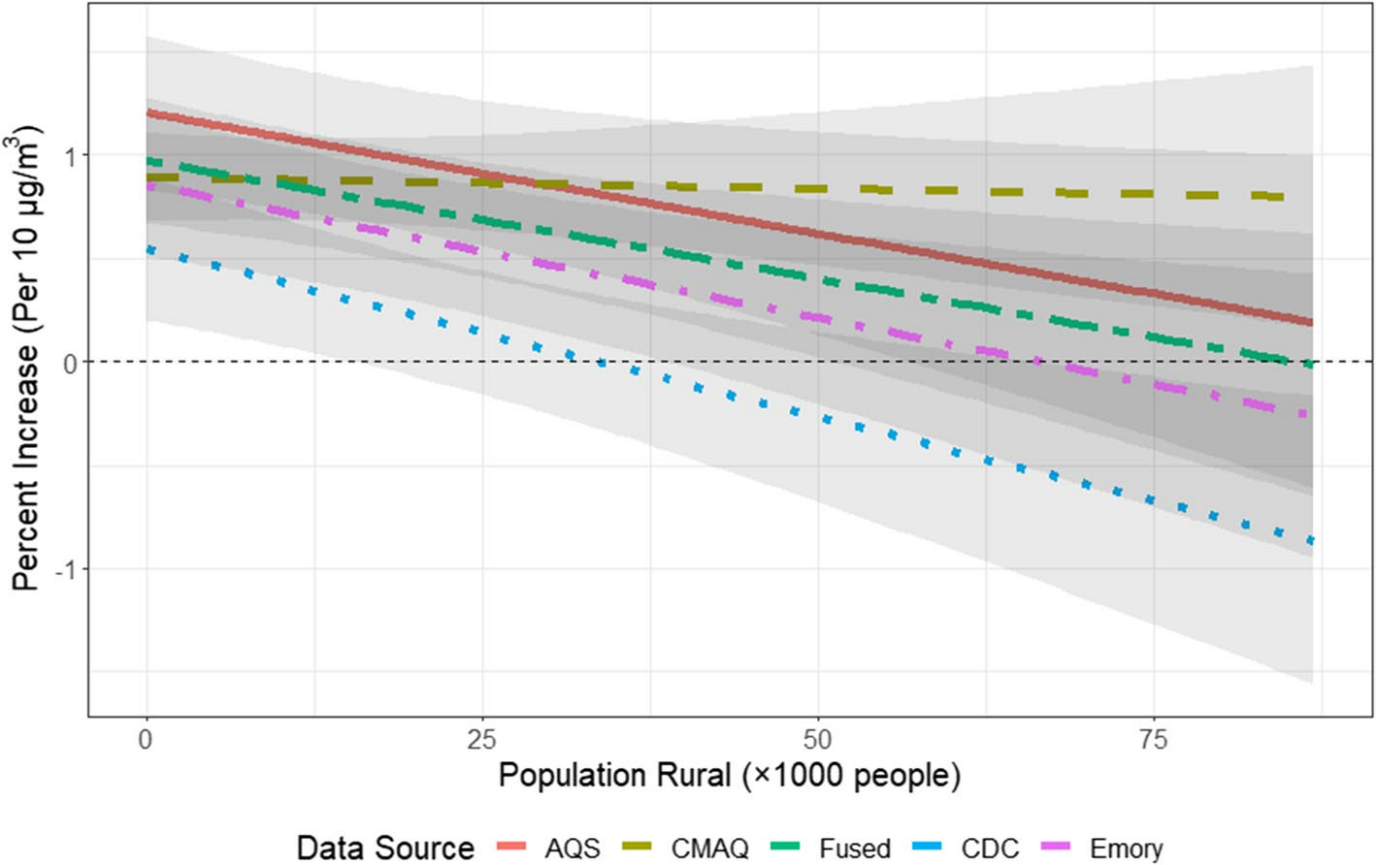
Spatial effect modification using all available data

Figure 4 shows the results assessing effect modification by rural population. Results are displayed as the percent increase in CVD admissions for a 10 µg/m^3^ increase in PM_2.5_ for each 1000-person increase in the rural population of each county. In all but the CMAQ model, we detected decreases in the effect estimates as rural population increased, i.e., the highest effect estimates were observed in urban areas.

## Discussion

Using five different sets of PM_2.5_ data spanning from 2002 to 2012, we investigated the relationship between daily PM_2.5_ and CVD hospital admissions in NYS, and found consistently harmful associations across all exposure metrics, albeit effect estimates quantitatively varied by a factor of almost four. In subsequent analyses, we explored potential spatial and temporal effect modification. We found higher effect estimates in the autumn and winter and higher effect estimates in more urban areas. These results were also largely consistent across exposure metrics.

There are a few papers in the literature that evaluate performance across different air pollution models, most of which focus on only one or two models. Bravo et al. compared PM_2.5_ predictions from a CMAQ simulation to that of ground-based monitors and found that CMAQ underestimated PM_2.5_, with substantial variations seasonally [28]. Lee et al. developed a space–time geostatistical kriging model to predict PM_2.5_ and compared these predictions to satellite-based PM_2.5_ estimates directly from AOD retrievals; they found that the kriging model provided more accurate estimates within 100 km of a monitoring station, while satellite estimates were more accurate for locations greater than 100 km from a monitoring station [29]. Jin et al. compared seven publicly available PM_2.5_ products over NYS from 2002 to 2012, including information from ground-based observations, remote sensing, and chemical transport models, and found that while the products differed in spatial patterns, all showed consistent decreases in PM_2.5_ over the observed time period [25]. A recent study by Kelly et al. comparing nine PM_2.5_ exposure models in the United States in 2011 found generally consistent PM_2.5_ concentrations but more variations at finer scales [30].

To date, most existing air pollution epidemiologic studies that assign exposure based on prediction models typically only use data from a single model to assign exposures [17, 18, 31]. We are only aware of a few existing epidemiologic studies that incorporate more than one exposure models. Weber et al. [32] conducted a case-crossover study over New York City from 2004–2006 looking at the association between short-term exposure to PM_2.5_ and heart failure, utilizing five different exposure models that combine air pollution monitors, aerosol optical depth (AOD), and CMAQ. They found that effect estimates across the models were similar. McGuinn et al. [33] investigated the association between long-term exposure to PM_2.5_ and cardiovascular disease using different exposure assessment methods. Utilizing station monitoring data, two CMAQ models, and two satellite-based models from 2002 to 2009 for a cohort of patients who had undergone a cardiac catherization residing in North Carolina, they found nearly equivalent results for all exposure assessment methods. Sellier et al. [34] used four different exposure models to estimate nitrogen dioxide (NO_2_) and particulate matter (PM) levels in two French metropolitan areas and explored their association with infant birthweight in a pregnancy cohort. They found consistent estimated health effects for the PM products, but less so for the NO_2_ products. Wang et al. [35] compared exposure estimates and associations for NO_2_ and various PM sizes based on predictions from a land-use regression and a dispersion model, and found that health effect estimates did not differ significantly. More recently, Gariazzo et al. [36] investigated the effects of long-term exposure to PM and NO_2_ using four exposure models in a large administrative cohort, and found consistent health effect estimates across the different exposure assessment models. Our work expands on this body of literature and assesses variability in acute CVD effect estimates from a time-series analysis using different existing exposure models.

In our main analysis, we found harmful associations across all models, although the confidence intervals of one model crossed the null. However, effect estimates fluctuated by a factor of four across the different exposure models. These differences are likely due to varying degrees of measurement error across the different models, as non-differential exposure measurement error in time series studies biases effect estimates towards the null [13]. The predictive accuracy of all these models likely varies in space and time in different ways, which could best explain the differences in our results. In the most comparable analyses (i.e., “AQS only” and “complete-case analyses”), the confidence intervals for all effect estimates widely overlap. While the results from the “complete-case analysis” may not be generalizable, because this analysis focuses on predominantly urban areas, it is the only analysis that allows direct comparison of the effect estimates across the five PM_2.5_ products. Therefore, we cannot conclude that these models truly yield different estimates.

In our secondary analysis of seasonal effect modification, we found higher effect estimates in the autumn and winter seasons. With the available information that we have, it is not possible to attribute this finding to varying impacts of exposure measurement error across seasons or to a biological mechanism. One possible explanation for these findings could be that all prediction models perform better during the autumn and winter months [25]; smaller amounts of exposure measurement error would result in de-attenuation of effect estimates. A second explanation could be that in NYS, estimated PM_2.5_ effects on CVD admissions are worse during fall and winter months. Existing literature has had mixed findings regarding this topic. Studies by Bell et al. and Hsu et al. that investigated the effects of PM_2.5_ on CVD morbidity found highest effect estimates in the winter [37–39]. On the other hand, studies by Peng et al. and Dai et al., who investigated the effects of PM_2.5_ and mortality, found higher effect estimates in the spring and summer [40, 41]. In our study, the higher effect estimates may indicate a higher contribution from more localized particles, such as traffic, during the colder months. Traffic has been consistently identified as a particularly toxic source of PM_2.5_, full of combustion products such as black carbon and heavy metals [42–44]. Since the mixing height in the winter is lower, this will likely result in higher near-surface PM_2.5_ concentrations even if emissions remain the same, as they dilute within a smaller near-surface volume.

In comparison to the results for seasonal effect modification, the results for effect modification by rural population were much more consistent: effect estimates were highest in urbanized areas, and decreased as rural population increased. The decrease is least pronounced in the CMAQ model, where the interaction term was not statistically significant, and most in the CDC model. Again, this finding could be due to two possible explanations. First, it is possible that all models except CMAQ have higher predictive accuracy in more densely populated areas. This would not be surprising, as AQS monitors are located near urban centers or more densely populated areas and all models except CMAQ were trained on or fused with PM_2.5_ concentrations measured at monitoring stations [25]. Given that our results for spatial effect modification were very similar across all models except CMAQ, we would expect these models to perform similarly in NYS counties with limited or no monitoring. Second, our findings of effect modification by urbanicity may indicate that particle composition in urban areas may be more toxic than that of rural areas, which is consistent with our interpretation of seasonality as discussed above. Similar findings have been found in the literature [45, 46], also likely because the distribution of potential effect modifiers, other than just PM_2.5_ composition, is different in urban versus rural areas.

Our study has several limitations. First and foremost, the results from our analyses may have some comparability issues. As mentioned previously, only 18 of 62 NYS counties included monitors reporting to the AQS database. Consequently, the results using all data available from each product are not directly comparable to each other. We ran additional analyses restricting to counties with monitoring sites and to no missingness across all exposure models. While these results are more directly comparable to each other, their overall generalizability is lower, as they reflect predominantly urban areas. Furthermore, in the analyses with restricted observations, the comparability problem still remains: even by looking at only counties with AQS monitors, oftentimes these counties may only include a single monitor, and its measurements are then uniformly assigned as the exposure for the entire county. In comparison, this is very different from the PM_2.5_ output we obtain from any of the exposure models that provide much finer spatial resolution.

Second, our analysis is conducted at the county level, which does not take full advantage of the fine-scale spatial resolution provided by the modeled exposure data. It is possible that the spatial aggregation may have introduced additional biases in the differences among the four exposure models used in our analyses, as the differences observed across the exposure models could be due to two sources: the true differences in predictions generated by each model at a specific location and any differences introduced as a result of the aggregation. However, our goal was to evaluate differences in estimated effects when using different exposure models in time series designs, which use aggregated health and exposure data. It is possible that any observed differences in effect estimates in our study would be different in other study designs, e.g., using individual-level data.

Third, we do not explore potential effects of lagged exposure to PM_2.5_ on CVD admissions in this study. A number of previous studies have found evidence for lagged effects for CVD related outcomes, mainly for exposure on the same and previous day of the CVD event (lag 0–1) and that of the same and previous 3 days combined (lag 0–3) [4, 47–49]. However, the goal of our analysis was to compare different exposure metrics and not to identify critical exposure windows, which we believe is beyond the scope of this study. Nonetheless, to facilitate comparison to other studies of PM_2.5_ and CVD outcomes, we include the results of lag 0–1 exposure for all PM_2.5_ products in the Supplement (Figure S3).

Lastly, none of the modeled PM_2.5_ data we used were perfect: each was built and optimized for different reasons and, thus, could overpredict in certain areas and underpredict in others. For example, CMAQ was originally designed to address regional air pollution problems across the United States, while the Emory product focuses on providing accurate PM_2.5_ predictions over NYS only. All PM_2.5_ products in this analysis (with the exception of CMAQ) utilized AQS monitors as part of the modeling process, which means that these PM_2.5_ products are likely to provide more accurate estimates near monitoring sites, i.e., mainly in urban areas. Furthermore, information on the predictive accuracy of these models is not always available, making it difficult to conduct a formal comparison of the different PM_2.5_ products beyond a qualitative assessment. Our previous work has attempted to evaluate these different PM_2.5_ products using three major criteria: resolution, availability, and accuracy. We found that no single product stood out for all three criteria, and the choice of PM_2.5_ product for the purposes of epidemiologic studies should depend on the research question of interest [25].

Nonetheless, our aim was not to identify the “best product” out of the ones examined. All models examined have nationwide coverage and our NYS results may not generalize to other states. Moreover, the choice of model should primarily depend on the study design and whether spatial vs. temporal contrasts are more important for each specific design and research question. Rather, we aimed to characterize the potential variability in estimated effect estimates in time-series analyses, using the NYS daily PM_2.5_ – CVD association as a case study. Based on our results, we feel comfortable to conclude that while the point effect estimates in our main analysis differ by as much as a factor of four, their corresponding confidence intervals were largely overlapping, and that different PM_2.5_ products do indeed reach the same conclusion. We also recognize that current health impacts assessments primarily use only point effect estimates from epidemiologic studies as inputs into calculations, and that the resulting conclusions of such assessments may differ drastically depending on the choice of the exposure–response function, which in turn depends on the choice of exposure model. Our findings highlight the importance of incorporating different sources of uncertainty in the exposure–response curves used in health impacts assessments, including uncertainty due to the choice of exposure model.

Our study has numerous strengths. We were able to investigate the sensitivity of estimated PM_2.5_ – CVD effects to exposure model choice in short-term epidemiologic studies. Given the increasing use of modeled air pollution data in health studies, our work is critical as it provides an example of how much estimated effects may vary across exposure models. In previous work, we evaluated how multiple PM_2.5_ products perform differently in a health impacts assessment [25]; this current work takes a step further by evaluating the impact of multiple PM_2.5_ products on the effect estimates that are used in such assessments. Finally, our findings send a strong public health message: increased PM_2.5_ exposure results in an increase in CVD hospitalizations, regardless of the choice of exposure model.

## Conclusions

In conclusion, we investigated the relationship between short-term PM_2.5_ exposure and cardiovascular admissions in NYS from 2002 – 2012 using five different PM_2.5_ products and found consistent, harmful associations regardless of exposure metric. However, uncertainty related with the exposure model selection is not captured in the individual estimated effects. Methods are needed for improved exposure assessment that minimize error and include uncertainty characterization and propagation into the health models [50].

## Supplementary Information

**Additional file 1: Table S1.** Descriptive statistics by season. **Table S2.** Descriptive statistics by quartiles of rural population. **Table S3.** Descriptive statistics for complete-case analysis. **Table S4.** Percent increase in daily CVD admissions and 95% confidence intervals (CI) per 10 µg/m^3^ for all PM_2.5_ products. **Table S5.** Quasi-Akaike’s Information Criterion (qAIC) for select seasonal and long-term trends. **Figure S1.** Percent increase in daily CVD admissions rates per interquartile range for all PM_2.5_ products. **Figure S2.** Percent increase in daily CVD admissions rates for subset excluding AQS. **Figure S3.** Percent increase in lag 0**–**1 CVD admissions rates per 10 µg/m^3^ for all PM_2.5_ products. **Figure S4.** Sensitivity analysis adding bank holiday as a potential confounder. **Figure S5.** Time series of average PM_2.5_ exposure estimates by PM_2.5_ product.

## Data Availability

The exposure data that support the findings of this study are available from the corresponding author on reasonable request. The health data that support the findings of this study are available from the New York State Department of Health but restrictions apply to the availability of these data, which were used under license for the current study, and so are not publicly available. Data are however available from the authors upon reasonable request and with permission of the New York State Department of Health.
