# Supplementary material for: Short-term PM2.5 and cardiovascular admissions in NY State: assessing sensitivity to exposure model choice
Source: Environ Health. 2021 Aug 23;20:93. doi: 10.1186/s12940-021-00782-3 (PMC8383435; doi:10.1186/s12940-021-00782-3)

***Supplemental material for: Short-term PM_2.5_ and cardiovascular admissions in NY State: assessing sensitivity of exposure model choice***

Authors: Mike Z. He^1^*^,2†^, Vivian Do^1^, Siliang Liu^1^, Patrick L. Kinney^3^, Arlene M. Fiore^4,5^, Xiaomeng Jin^6^, Nicholas DeFelice^2^, Jianzhao Bi^7^, Yang Liu^8^, Tabassum Z. Insaf^9,10^, Marianthi-Anna Kioumourtzoglou^1^

**Author Affiliations:**

^1^ Department of Environmental Health Sciences, Columbia University Mailman School of Public Health, New York, NY, USA

^2^ Department of Environmental Medicine and Public Health, Icahn School of Medicine at Mount Sinai, New York, NY, USA

^3^ Department of Environmental Health, Boston University School of Public Health, Boston, MA, USA

^4^ Department of Earth and Environmental Sciences, Columbia University, New York, NY, USA

^5^ Lamont-Doherty Earth Observatory, Columbia University, Palisades, NY, USA

^6^ Department of Chemistry, University of California, Berkeley, Berkeley, CA, USA

^7^ Department of Environmental & Occupational Health Sciences, University of Washington School of Public Health, Seattle, WA, USA

^8^ Gangarosa Department of Environmental Health, Emory University, Rollins School of Public Health, Atlanta, GA, USA

^9^ New York State Department of Health, Albany, NY, USA

^10^ School of Public Health, University at Albany, Rensselaer, NY, USA

## ***** At the time the work was conducted.

† **Corresponding Author:** Mike He, Department of Environmental Medicine and Public Health, Icahn School of Medicine at Mount Sinai, One Gustave L. Levy Place, Box 1057, New York, NY, USA 10029 E-mail: mike.he@mssm.edu

**Table of Contents**

Table S1. Descriptive statistics by season……………………………………………………………...3

Table S2. Descriptive statistics by quartiles of rural population…………………………….…….….4

Table S3. Descriptive statistics for complete-case analysis………………………………………….5

Table S4. Percent increase in daily CVD admissions and 95% confidence intervals (CI) per 10 µg/m^3^ for all PM_2.5_ products ………………………………………………………………….………….5

Table S5. Quasi-Akaike’s Information Criterion (qAIC) for select seasonal and long-term trends ………………………………………………………………………………………………………...……5

Figure S1. Percent increase in daily CVD admissions rates per interquartile range for all PM_2.5_ products …………………………………………………………………………………………...………6

Figure S2. Percent increase in daily CVD admissions rates for subset excluding AQS …………………………………………………...…………………………………………………………7

Figure S3. Percent increase in lag 0**–**1 CVD admissions rates per 10 µg/m^3^ for all PM_2.5_ products…………………………………………………………………………………………………....8

Figure S4. Sensitivity analysis adding bank holiday as a potential confounder……………………9

Figure S5. Time series of average PM_2.5_ exposure estimates by PM_2.5_ product……….…....…….10

**Table S1. Descriptive statistics by season for all counties included in analyses, unless otherwise noted (2002 – 2012).**

| **Variable** | **Mean** | **Min** | **25%** | **50%** | **75%** | **Max** | **% Missing** |
| --- | --- | --- | --- | --- | --- | --- | --- |
| *Spring (1,012 days)* |  |  |  |  |  |  |  |
| Daily CVD admission counts per county | 7.1 | 0.0 | 1.0 | 2.0 | 6.0 | 115.0 | 0.7 |
| PM_2.5_ (µg/m^3^)  AQS^a^  CMAQ  Fused  CDC^b^  Emory | 9.4  8.2  8.5  8.7  8.2 | 0.1  0.0  0.3  0.0  0.4 | 5.5  4.0  5.1  5.4  4.3 | 8.2  6.6  7.4  7.7  6.5 | 12.0  10.5  10.7  10.7  10.1 | 68.9  88.2  51.2  58.7  53.1 | 75.8 0.0 0.4 18.2 1.7 |
| Mean temperature (°C) | 7.6 | -22.1 | 2.8 | 7.9 | 12.6 | 27.2 | 0.0 |
| Relative humidity (%) | 75.8 | 34.5 | 68.3 | 77.4 | 84.9 | 98.4 | 0.0 |
| *Summer (1,012 days)* |  |  |  |  |  |  |  |
| Daily CVD admission counts per county | 6.7 | 0.0 | 1.0 | 2.0 | 5.0 | 102.0 | 0.8 |
| PM_2.5_ (µg/m^3^)  AQS^a^  CMAQ  Fused  CDC^b^  Emory | 13.0 7.5 12.2 12.2 11.0 | 0.0 0.1 0.3 0.0 1.0 | 6.2 3.7 6.8 7.8 5.3 | 10.6 6.0 10.2 10.7 8.8 | 17.6 9.9 15.6 15.4 15.0 | 97.1 53.4 84.1 58.7 81.3 | 75.8 0.0 0.3 18.2 1.5 |
| Mean temperature (°C) | 20.4 | 5.9 | 18.2 | 20.6 | 22.9 | 31.5 | 0.0 |
| Relative humidity (%) | 79.2 | 49.6 | 74.5 | 79.5 | 84.2 | 98.2 | 0.0 |
| *Autumn (1,001 days)* |  |  |  |  |  |  |  |
| Daily CVD admission counts per county | 6.7 | 0.0 | 1.0 | 2.0 | 5.0 | 115.0 | 0.8 |
| PM_2.5_ (µg/m^3^)  AQS^a^  CMAQ  Fused  CDC^b^  Emory | 9.5 8.3 8.4 8.0 6.9 | 0.0 0.0 0.3 0.0 0.7 | 5.2 3.7 4.8 4.8 3.6 | 8.1 6.4 7.1 7.1 5.5 | 12.2 10.9 10.7 9.9 8.7 | 51.5 98.2  64.8 51.3 51.2 | 75.7 0.0 0.3 18.2 1.9 |
| Mean temperature (°C) | 10.9 | -9.4 | 5.9 | 11.0 | 16.3 | 27.8 | 0.0 |
| Relative humidity (%) | 79.3 | 24.6 | 73.5 | 79.6 | 86.0 | 98.3 | 0.0 |
| *Winter (993 days)* |  |  |  |  |  |  |  |
| Daily CVD admission counts per county | 6.8 | 0.0 | 1.0 | 2.0 | 5.0 | 113.0 | 0.8 |
| PM_2.5_ (µg/m^3^)  AQS^a^  CMAQ  Fused  CDC^b^  Emory | 10.9 10.9 9.8 9.2 7.8 | 0.2 0.2 1.2 0.0 1.3 | 7.2 5.7 6.0 4.7 4.9 | 9.8 9.1 8.7 7.6 6.7 | 13.4 13.9 12.4 12.0 9.5 | 44.8 93.6 99.7 56.5 47.5 | 75.7 0.0 0.4 18.2 3.5 |
| Mean temperature (°C) | -3.0 | -25.3 | -6.3 | -2.6 | 0.5 | 16.4 | 0.0 |
| Relative humidity (%) | 83.1 | 27.4 | 78.9 | 84.9 | 89.5 | 100.9 | 0.0 |

^a^ Monitoring sites were only available in 18 of the 62 counties in NYS; ^b^ Data were only available for 2003 – 2011.

**Table S2. Descriptive statistics by quartiles of rural population (least to most rural) across all counties in the study, unless otherwise noted (2002 – 2012).**

| **Variable** | **Mean** | **Min** | **25%** | **50%** | **75%** | **Max** | **% Missing** |
| --- | --- | --- | --- | --- | --- | --- | --- |
| *1^st^ quartile (0 – 21,800)* |  |  |  |  |  |  |  |
| Daily CVD admission counts per county | 14.7 | 0.0 | 1.0 | 3.0 | 28.0 | 115.0 | 2.5 |
| PM_2.5_ (µg/m^3^)  AQS^a^  CMAQ  Fused  CDC^b^  Emory | 12.5 11.0 10.7 10.3 9.3 | 0.5 0.0 0.3  0.0 0.4 | 7.4 5.0 6.0 5.8 4.9 | 10.8 8.4 10.7 8.6 7.4 | 15.6 14.1 13.6 13.2 11.8 | 86.1 98.3 84.1 58.7 81.3 | 62.3 0.0 0.0 18.2 2.1 |
| Mean temperature (°C) | 10.1 | -25.0 | 1.9 | 10.5 | 18.9 | 31.5 | 0.0 |
| Relative humidity (%) | 78.4 | 24.6 | 72.2 | 79.4 | 86.0 | 99.4 | 0.0 |
| *2^nd^ quartile (21,801 – 36,800)* |  |  |  |  |  |  |  |
| Daily CVD admission counts per county | 2.5 | 0.0 | 0.0 | 1.0 | 2.0 | 43.0 | 0.4 |
| PM_2.5_ (µg/m^3^)  AQS^a^  CMAQ  Fused  CDC^b^  Emory | 7.9 7.7 9.1 9.2 7.4 | 0.0 0.0 0.3 0.0 0.7 | 4.0 3.7 5.2 5.4 3.9 | 6.3 6.2 7.7 8.0 5.8 | 10.0 10.1 11.4 11.6 9.1 | 97.1 65.2 76.1 49.9  78.7 | 87.1 0.0 0.0 18.2 2.2 |
| Mean temperature (°C) | 8.4 | -25.3 | 0.0 | 8.8 | 17.6 | 30.3 | 0.0 |
| Relative humidity (%) | 79.3 | 32.4 | 73.9 | 80.4 | 86.3 | 100.1 | 0.0 |
| *3^rd^ quartile (36,801 – 52,300)* |  |  |  |  |  |  |  |
| Daily CVD admission counts per county | 4.9 | 0.0 | 1.0 | 2.0 | 4.0 | 75.0 | 0.0 |
| PM_2.5_ (µg/m^3^)  AQS^a^  CMAQ  Fused  CDC^b^  Emory | 10.1 8.2 9.7 9.3 8.1 | 0.4 0.0 0.3 0.0 0.7 | 6.0 4.2 5.6 5.5 4.4 | 8.6 6.8 8.3 8.2 6.5 | 12.6 10.8 12.2 11.9 10.0 | 78.8 68.2 99.7 55.5 70.5 | 76.4 0.0 1.2 18.2 2.1 |
| Mean temperature (°C) | 8.7 | -21.3 | 0.6 | 9.1 | 17.8 | 29.8 | 0.0 |
| Relative humidity (%) | 80.1 | 26.2 | 74.9 | 81.2 | 86.9 | 100.9 | 0.0 |
| *4^th^ quartile (52,301 – 86,400)* |  |  |  |  |  |  |  |
| Daily CVD admission counts per county | 4.9 | 0.0 | 2.0 | 3.0 | 6.0 | 43.0 | 0.0 |
| PM_2.5_ (µg/m^3^)  AQS^a^  CMAQ  Fused  CDC^b^  Emory | 9.8 8.0 9.5 9.2 7.8 | 0.0 0.0 0.3 0.0 0.9 | 5.5 3.9 5.5 5.5 4.2 | 8.4 6.5 8.0 8.1 6.2 | 12.4 10.6 11.8 11.6 9.6 | 55.8 61.1 74.3 47.9 72.2 | 78.0 0.0 0.0 18.2 2.2 |
| Mean temperature (°C) | 8.8 | -24.0 | 0.5 | 9.2 | 18.1 | 30.5 | 0.0 |
| Relative humidity (%) | 79.5 | 27.4 | 74.1 | 80.7 | 86.5 | 99.7 | 0.0 |

^a^ Monitoring sites were only available in 18 of the 62 counties in NYS; ^b^ Data were only available for 2003 – 2011.

**Table S3. Descriptive statistics for complete-case analysis in 18 counties (3,234 days).**

| **Variable** | **Mean** | **Min** | **25%** | **50%** | **75%** | **Max** |
| --- | --- | --- | --- | --- | --- | --- |
| Daily CVD admission counts per county | 20.9 | 0.0 | 5.0 | 15.0 | 33.0 | 115.0 |
| PM_2.5_ (µg/m^3^)  AQS  CMAQ  Fused  CDC  Emory | 10.8 8.4 11.0 10.6 9.7 | 0.0 0.0 0.3 0.0 1.0 | 6.1 5.0 6.1 6.0 5.2 | 9.1 8.4 9.2 8.9 7.9 | 13.8 14.0 14.0 13.5 12.5 | 76.0 98.2 99.7 58.7 56.1 |
| Mean temperature (°C) | 10.2 | -24.8 | 2.3 | 10.6 | 18.9 | 31.5 |
| Relative humidity (%) | 78.9 | 30.1 | 72.8 | 80.1 | 86.6 | 99.3 |

**Table S4.** **Percent increase in daily CVD admissions and 95% confidence intervals (CI) per 10 µg/m^3^ for all PM_2.5_ products.**

|  | Analysis Type | | | | | |
| --- | --- | --- | --- | --- | --- | --- |
| PM_2.5_ Source | All Data | | AQS Only | | Complete Case | |
|  | Estimate | 95% CI | Estimate | 95% CI | Estimate | 95% CI |
| AQS | NA | NA | 0.96 | (0.62, 1.30) | 0.83 | (0.45, 1.21) |
| CMAQ | 0.88 | (0.68, 1.08) | 0.93 | (0.71, 1.16) | 0.96 | (0.70, 1.21) |
| Fused | 0.78 | (0.51, 1.05) | 0.89 | (0.58, 1.20) | 0.78 | (0.43, 1.13) |
| CDC | 0.23 | (-0.06, 0.53) | 0.49 | (0.14, 0.84) | 0.50 | (0.16, 0.85) |
| Emory | 0.62 | (0.31, 0.92) | 0.79 | (0.44, 1.15) | 0.81 | (0.42, 1.21) |

**Table S5.** **Quasi-Akaike’s Information Criterion (qAIC) for select seasonal and long-term trends. qAIC Values shown here are for when the Fused PM_2.5_ product is used as the exposure of interest.**

| Degrees of Freedom | qAIC |
| --- | --- |
| 4 | **859874.2** |
| 5 | 860076.2 |
| 6 | 860136.8 |
| 7 | 860268.3 |

**Figure S1. Percent increase in daily CVD admissions rates per interquartile range for all PM_2.5_ products.**


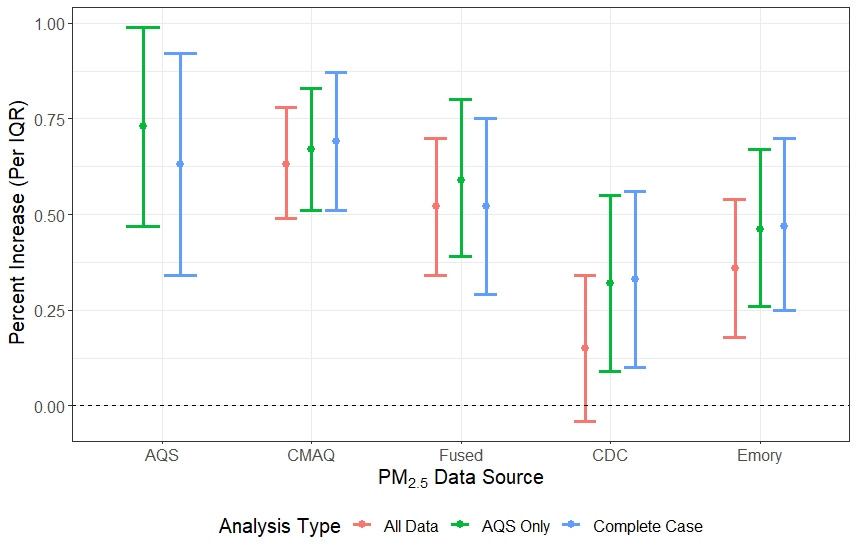


**Figure S2.** **Percent increase in daily CVD admissions rates for the subset excluding AQS. Note that the AQS only results are presented here for reference.**


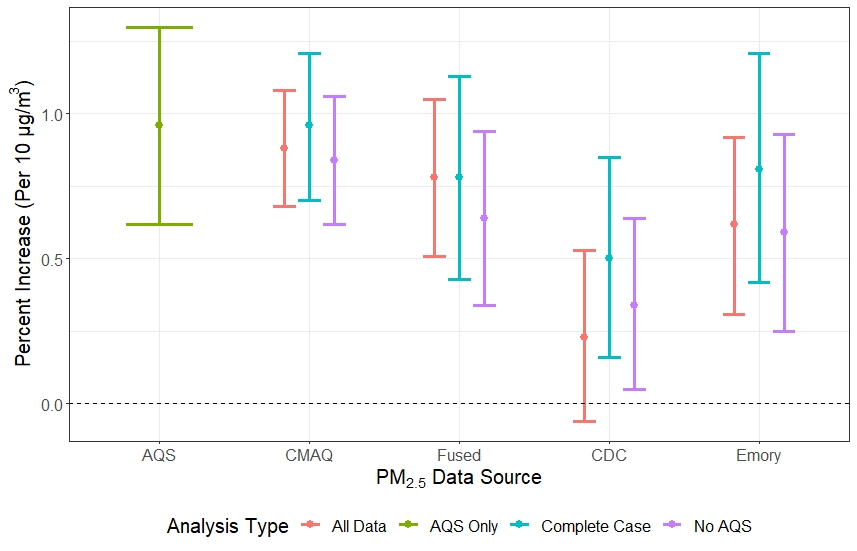


**Figure S3.** **Percent increase in lag 0****–1 CVD admissions rates per 10 µg/m^3^ for all PM_2.5_ products.**

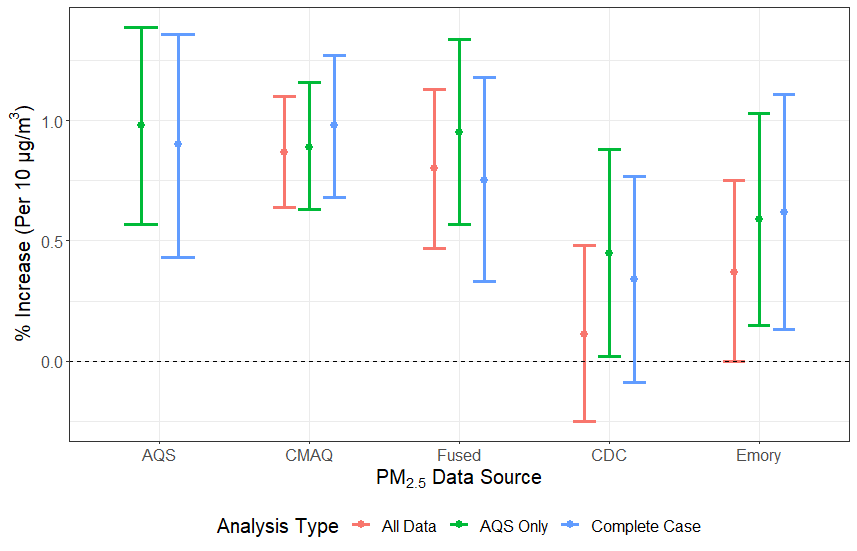


**Figure S4.** **Sensitivity analysis adding bank holiday as a potential confounder. Here, “Main Analysis” refers to the analysis for which all available data is used.**

**
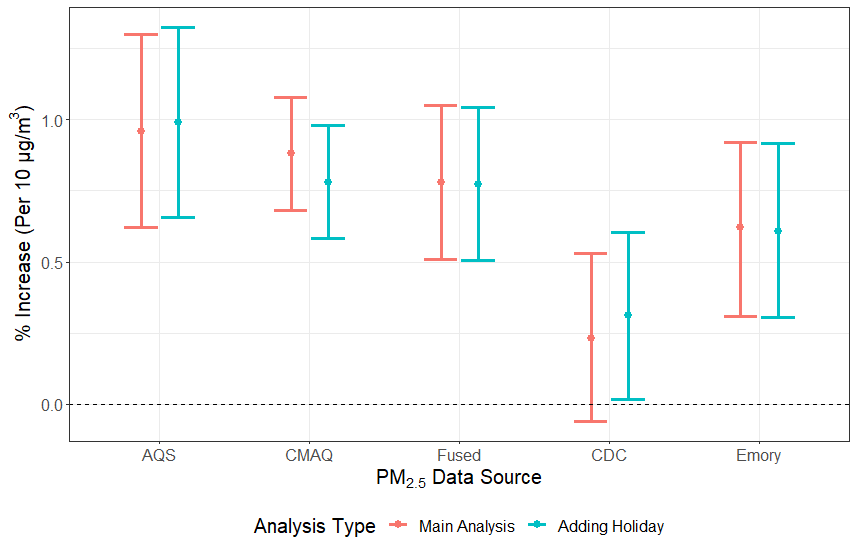
**

**Figure S5.** **Time series of average PM_2.5_ exposure estimates by PM_2.5_ product.**


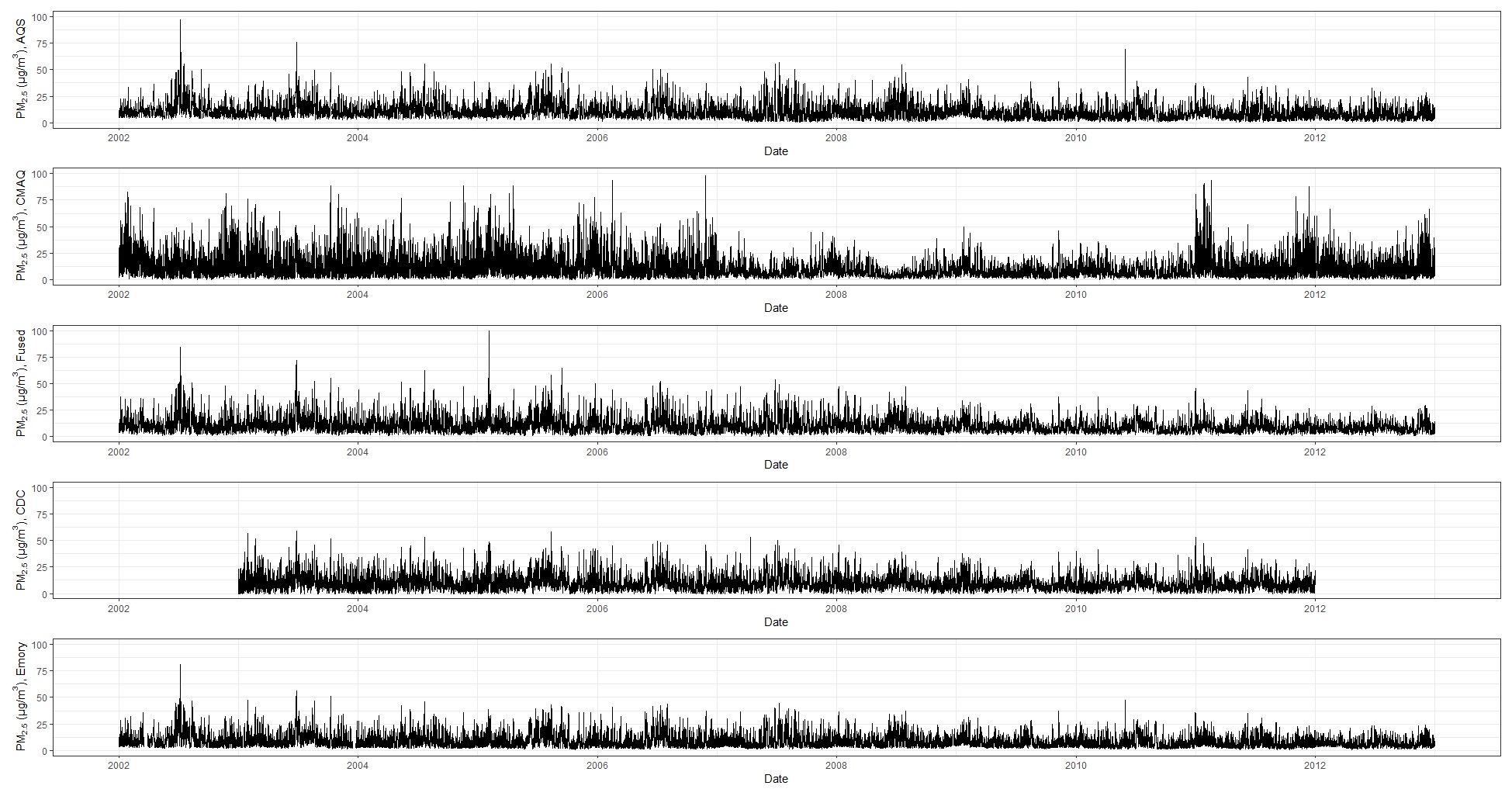

Supplement: Supplementary file 1 — Additional file 1: Table S1. Descriptive statistics by season. Table S2. Descriptive statistics by quartiles of rural population. Table S3. Descriptive statistics for complete-case analysis. Table S4. Percent increase in daily CVD admissions and 95% confidence intervals (CI) per 10 µg/m3 for all PM2.5 products. Table S5. Quasi-Akaike’s Information Criterion (qAIC) for select seasonal and long-term trends. Figure S1. Percent increase in daily CVD admissions rates per interquartile range for all PM2.5 products. Figure S2. Percent increase in daily CVD admissions rates for subset excluding AQS. Figure S3. Percent increase in lag 0–1 CVD admissions rates per 10 µg/m3 for all PM2.5 products. Figure S4. Sensitivity analysis adding bank holiday as a potential confounder. Figure S5. Time series of average PM2.5 exposure estimates by PM2.5 product. [file 12940_2021_782_MOESM1_ESM.docx]
